# Supplementary material for: Alteration of Gut Immunity and Microbiome in Mixed Granulocytic Asthma
Source: Biomedicines. 2022 Nov 16;10(11):2946. doi: 10.3390/biomedicines10112946 (PMC9687559; doi:10.3390/biomedicines10112946)
Supplement: Supplementary file 1 [file biomedicines-10-02946-s001.zip › biomedicines-1972930-supplementary.pdf]

Supplementary Data

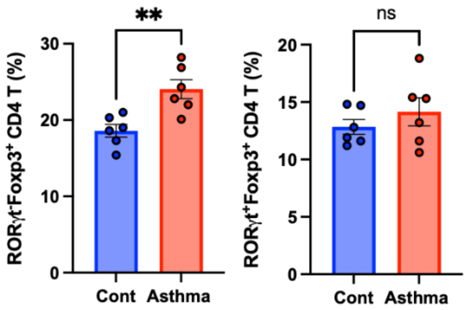

Supplementary Figure S1. Frequency of RORγt$^-$Foxp3$^+$ CD4 T cell and RORγt$^+$Foxp3$^+$ CD4 T cell subsets gated on CD3 in the colon.

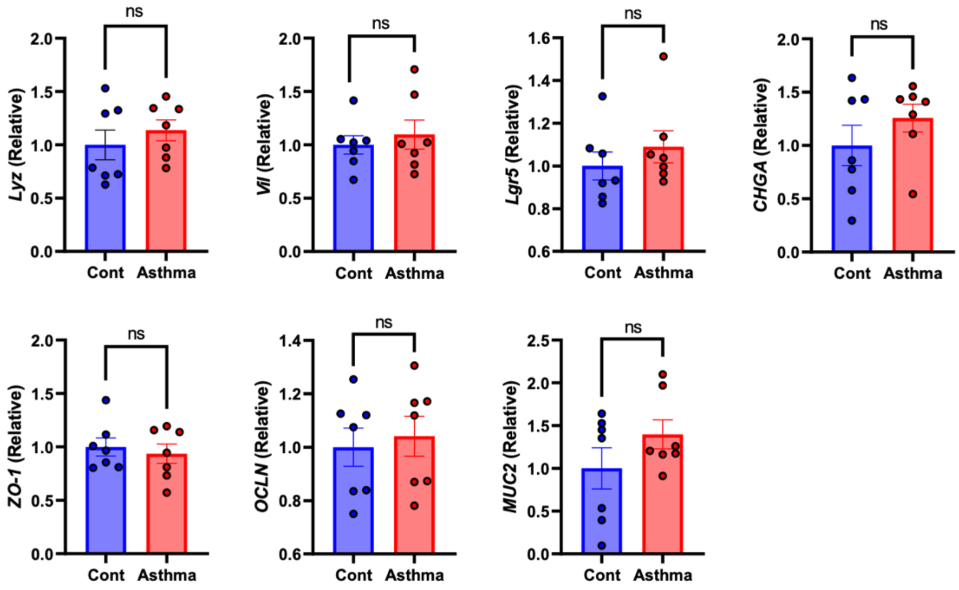

Supplementary Figure S2. Relative expression of genes for intestinal epithelial cell markers and tight junction; lysozyme (Lyz), villin (Vil), leucine-rich repeat-containing G-protein coupled receptor 5 (Lgr5), chromogranin A (CHGA), zonula occludens-1 (ZO-1), occluding (OCLN), and mucin 2 (MUC2).
